# Mr. Ring, on Vaccine Inoculation

**Published:** 1803-04-01

**Authors:** John Ring

**Affiliations:** New Street, Hanover Square


### *To the Editors of the Medical and Physical Journal.*

Gentlemen,

A CASE lately occurred, which proves the necessity of a little more care in conducting Vaccine Inoculation, than has hitherto been observed by practitioners in general.

A child of Mr. Coxe, in St. George's Street, near the Asylum, having been inoculated for the Cow-pox by a Surgeon in the country, Mr. Coxe desired me to examine the arm in which the operation had been performed. No scar appearing, I inoculated the child again, and produced a perfect Vaccine pustule.

Had not this patient been inoculated a second time, a favourable opportunity of depreciating Vaccination would in all probability have been afforded to the enemies of that practice.

It must give great pleasure to the friends of Vaccination to hear, that it is introduced into Barbadoes. For this information I am indebted to Mr. Dale of Liverpool, who first practised Vaccine Inoculation in that town, and furnished a Surgeon going to Barbadoes with the matter, which proved successful.

Some Vaccine virus which I collected, was sent by the Rev. Mr. Smith, of Camberwell, to Revel; where it succeeded. Some months ago, Dr. Jenner received from the Surgeon Major of Revel, an account, that from this source Vaccination was rapidly spreading through that part of the Russian dominions, and had already been practised upon many thousands.

I have the authority of a nobleman, who resided as his Majesty's Ambassador at the court of Petersburgh, for asserting, that this salutary practice seemed to be more generally adopted in Russia than in England. This, I trust, will excite emulation in the breasts of our countrymen, and add a fresh stimulus to their exertions.

On

On a former occasion I recommended tooth-picks, for the preservation and conveyance of matter. Would medical practitioners preserve it in this, or some similar mode, they would always have a supply in case of emergency. It is well known, that it retains its virtue more than three months; and in one instance, Dr. De Carro successfully used a thread six months and three days old; which he had carried in his pocket, wrapped up in a letter, from the time of its reception.

As a proof of the superiority of the method of preserving Vaccine virus on a tooth-pick, over those in common use, I shall copy part of a letter which I lately received from Dr. Tierney, of Brighton, who is well known to have been an early correspondent of Dr. Jenner, and who, since that period, had a considerable share in introducing it to the notice of the Professors in the University of Edinburgh, and published an Inaugural Thesis upon the subject at Glasgow.

"I shall be much obliged to you for a little more Vaccine virus on a tooth-pick, as you sent it before. I am much pleased with your mode. It answers extremely well. I have before sent Virus to Ireland several times; but, from some cause or other, it has not succeeded; until, some time back, I sent some after your manner, on a piece of quill, shaped like a tooth-pick.

"With this, some near relatives of my own have been inoculated; and it has succeeded completely. The account which I received will show, how many errors have been committed from ignorance of the true disease.

"When the medical men saw the pustule produced by this virus, they unanimously declared,—'That although they had been dabbling in the Inoculation for the Cow-pock THREE YEARS, this was the first case of TRUE COW-POCK they had witnessed.'—Such errors are not uncommon. Within a very short time, a female in this place, who said she had been inoculated for the Cow-pock two years ago, has had the Small-pox. On inquiry, it turns out, that she had only a slight inflammation of the part, which has left no mark behind. Every day's experience proves more forcibly the necessity of caution and attention in this practice."

Such have been the errors committed at Brighton, that Dr. Tierney will find it a difficult task to render the practice popular there; much, however, may be expected from his talents and exertions.

Instead of tooth-picks, I now use a small bit of ivory, formed formed like the tooth of a comb, and pointed like a lancet. The idea of these simple instruments was suggested by an ivory lancet which Dr. De Carro, of Vienna, did me the houour to send me, together with the second edition of his excellent publication. That instrument is extremely neat, and well adapted for carrying in the pocket. It is fixed in a handle of mahogany, and screwed in a case of the same. Were the whole made of ivory, it would be stronger.

I have directed the small instrument above described to be made by Mr. Jackson, a comb-maker in High-street, Bloomsbury. I call it a Vaccinator. Public institutions, and private practitioners, who have a great demand for Cow-pock matter, will find this information useful. When few persons are to be supplied, lancets made of ivory, platina, or gold, may be employed; but when many thousands of practitioners are wanting a supply every year, cheaper articles must be discovered.

Would every person who inoculates, charge some of these Vaccinators well, with matter taken at an early period, and keep them wrapped up in paper, or enclosed in the barrel of a quill, they would furnish him with a constant supply, and the propagation of the practice would be greatly promoted. The quill may be stopped with white wax: the heat necessary to melt sealing wax might decompose the matter.

It is proper to remind Inoculators, that when those instruments are employed, a puncture should first be made with a lancet; then the point of the Vaccinator, armed with the virus, is to be introduced, and held in the part for the space of half a minute or more. Afterwards the flat surfaces of the instrument are to be repeatedly wiped on the puncture.

One Vaccinator, well charged, may serve for inoculating more than one person.

These hints will tend to facilitate a practice, on which the preservation of human life so much depends. I cannot conclude this letter without congratulating you on the establishment of a Society, which intends to supply medical practitioners with Cow-pock matter gratis.

It is true, that by the sale of matter, a Vaccine Institution might be enabled to support, and even to enrich itself, without subscriptions; and in the course of a few years, we might see the edifice devoted to that purpose rise, like a phœnix, from its own ashes, with an inscription tion paying the same compliment to Vaccina, as was paid to Augustus at Rome, *Invenit lateritiam, marmoream reliquit.* I am, &c.

JOHN RING.

*New Street, Hanover Square. March* 20, 1803.

P. S. Since the foregoing was written, Dr. Jenner has done me the honour to call on me, and to communicate the agreeable and important intelligence that Vaccine Inoculation has at length succeeded in India. This information he received from Dr. De Carro, who sent the matter by way of Bassora. It is confirmed by a gentleman just arrived from India, whom Dr. Jenner saw soon after he had read Dr. De Carro's letter.

